# Delayed wound healing after tooth extraction and self-reported kyphosis in Japanese men and women

**DOI:** 10.1038/srep36309

**Published:** 2016-11-16

**Authors:** Akira Taguchi, Mikio Kamimura, Yukio Nakamura, Noriyuki Sugino, Akira Ichinose, Hisayoshi Maezumi, Takashi Fukuzawa, Ryouhei Ashizawa, Kenji Takahara, Susumu Gushiken, Keijiro Mukaiyama, Shota Ikegami, Shigeharu Uchiyama, Hiroyuki Kato

**Affiliations:** 1 Department of Oral and Maxillofacial Radiology, School of Dentistry, Matsumoto Dental University, 1780 Gobara, Hirooka, Shiojiri, Nagano 399-0781, Japan; 2 Center of Osteoporosis and Spinal Disorders, Kamimura Orthopedic Clinic, 595-17 Ippommatsu, Kotobukitoyooka, Matsumoto, Nagano 399-0021, Japan; 3 Department of Orthopedic Surgery, Showa-Inan General Hospital, Akaho 3230, Komagane 399-4117, Japan; 4 Department of Orthopedic Surgery, Shinshu University School of Medicine, 33-1-1 Asahi, Matsumoto, Nagano 390-8621, Japan; 5 Department of Orthopedic Surgery, Ichise Hospital, 4824 Nishitakano-cho, Shimosuwa, Suwa-gun, Nagano 393-0087, Japan; 6 Maezumi Orthopedic Clinic, 8263-1 Hodaka, Azumino, Nagano 399-8303, Japan; 7 Department of Orthopedic Surgery, Shiojiri Hospital, 6-4-36 Daimon, Shiojiri, Nagano 399-0731, Japan; 8 Ashizawa Orthopedic Clinic, 12205-2 Nakaminowa, Minowacho, Kamiina-gun, Nagano 399-4601 Japan; 9 Takahara Clinic, 5586-2, Minami-Minowa, Kamiina-gun, Nagano, 399-4511 Japan; 10 Department of Surgery, Matsumoto Kyoritsu Hospital, 9-26 Habaue, Matsumoto, Nagano 390-8505, Japan; 11 Department of Orthopedic Surgery, North Alps Medical Center Azumi Hospital, 3207-1 Oaza-Ikeda, Ikeda, Kitaazumi-gun, Nagano 399-8695, Japan

## Abstract

It is unclear whether osteoporosis itself is a main risk factor for delayed wound healing after tooth extraction in humans. In this study, we evaluated the association between experience of delayed wound healing after last tooth extraction and self-reported kyphosis, with the possibility of having vertebral fractures, in Japanese patients. Among the 1,504 patients who responded to the structured questionnaire survey, 518 patients (134 men and 384 women) aged 55–97 years finally participated in this study. Patients who self-reported mild-moderate kyphosis were more likely to have problematic delayed wound healing after last tooth extraction than those who reported severe kyphosis (odds ratio [OR] 4.98; 95% confidence interval [CI], 1.86–13.38 and OR 2.30; 95% CI, 0.52–10.22, respectively) (p for trend = 0.005). Japanese patients with vertebral fractures may have a higher risk of having problematic delayed wound healing after tooth extraction.

Marx and Ruggiero *et al*. reported that Bisphosphonates (BP) treatment may increase the risk of osteonecrosis of the jaws (ONJ) in osteoporosis (OP) and oncology patients^1^,^2^, and the latter defined delayed wound healing after tooth extraction as ONJ^2^. Thus, views on the use of BPs for patients with OP vary among physicians, dentists, and patients. In 2015, the incidence of ONJ in OP patients was reported to be 0.001–0.01%, which may be only slightly higher than the frequency observed in the general population^3^. However, because of the paucity of prospective cohort data, it is difficult to accurately evaluate ONJ incidence^3^. The number of reports on ONJ increased until 2010, whereupon it plateaued. Conversely, numerous reports on BP-related ONJ (BRONJ) have been published since 2015, which includes international consensus report of ONJ^3^. Also, there have been many reports on the ONJ potentially caused by denosumab which inhibits bone metabolism like BP recently.

The diagnostic guideline of chronic disease is based on the threshold of surrogate markers that show increased risks of disease development as a complication. OP has been defined as “a skeletal disorder characterized by compromised bone strength, predisposing a person to increased risk of fracture”^4^. The surrogate marker for OP is bone mineral density (BMD), and BMD values with presence of high fracture risk are the diagnostic guideline of OP. According to the treatment recommendations of the National Osteoporosis Foundation (NOF), initial pharmacologic treatment factors could be hip or vertebral (clinical or asymptomatic) fractures^5^. Thus, the most apparent diagnostic evidence of OP is the complication of fragile fracture.

Several reports suggested that despite the high prevalence of vertebral fracture, many vertebral fractures remain undiagnosed. Because vertebral fractures are diagnosed using radiography, even patients without factures must be exposed to radiation^6^,^7^. Therefore, it is important to explore alternatives to radiography for diagnosing asymptomatic vertebral fracture, especially when considering large number of these patients^8^.

A recent questionnaire-based survey by the Japanese Society for Oral and Maxillofacial Surgeons indicated the rapid increase of ONJ (approximately 20-fold for patients both using and not using BP) in Japan when they compared the number of ONJ cases reported in 2006–2008 with that in 2011–2013 (unpublished data). BPs have been approved in Japan since 2001. ONJ overdiagnosis may occur because ONJ is broadly recognized among the dentists who may not be familiar with ONJ and likely to overdiagnose it.

Until recently, a large number of studies have addressed the potential role of BPs on jaw bones. However, there has been no strict control group with respect to OP characteristics against the patient’s group. Furthermore, the pathophysiology of ONJ is not well understood. Tanoue *et al*. recently demonstrated that bone formation was not impeded by short-term alendoronate treatment but was enhanced during wound healing after tooth extraction^9^. They concluded that alendoronate did not alter bone fill in extraction sockets. Additionally, some studies including randomized, placebo-controlled trials suggested that oral BP treatment may improve clinical outcomes of non-surgical periodontal therapy^10^,^11^. These results suggested that OP itself may be a main risk factor for delayed wound healing after tooth extraction. Therefore, both infection and delayed wound healing after tooth extraction may lead to an increased risk of prolonged bone repair and osteomyelitis of the jaws in OP patients.

Some studies have reported OP itself as a risk factor of ONJ^3^,^12^. However, to our knowledge, no previous study has reported the association between OP and delayed wound healing after tooth extraction. Approximately two thirds of women with radiographic evidence of vertebral fracture are unaware of the fracture^13^. However, we recently demonstrated that a self-reported kyphosis may be a useful marker for identifying postmenopausal women with asymptomatic vertebral fractures^14^. Therefore, in this study, we evaluated the association between self-reported experience of delayed wound healing after tooth extraction and self-reported kyphosis, which represents the potential presence of vertebral fractures, in Japanese men and women.

## Results

### Participants

During the study period, a total of 1,504 patients from 8 clinics or hospitals responded to a structured questionnaire with written informed consent. Of these, 617 patients who had missing or unclear responses in their questionnaires were excluded from this study. Of the remaining 887 patients, 369 patients who were treated using OP medications before their last tooth extractions were also excluded. Finally, 518 patients (134 men and 384 women) aged 55–97 years participated in this study. Patient characteristics are shown in Table 1. Regarding the time of last tooth extraction, 102 (19.7%) patients had undergone last tooth extraction <1 year previously, 69 (13.3%) patients between 1 and 3 years previously, and 347 (67.0%) patients >3 years previously. Three, three, and 25 patients experienced delayed wound healing after extraction, respectively.

### Association between delayed wound healing and self-reported kyphosis

ANOVA revealed significant differences in age (p = 0.011), height (p = 0.012), weight (p = 0.001), and number of teeth lost throughout the patient’s lifetime (p = 0.028) among the three self-reported kyphosis categories (Table 2). Additionally, self-reported kyphosis category was significantly associated with a self-reported periodontal condition (p = 0.040), history of smoking (p = 0.004) and delayed wound healing after last tooth extraction (p = 0.002). However, there was no significant association between time of last tooth extraction and self-reported kyphosis category (p = 0.703).

Stepwise logistic regression analysis with forward selection revealed that the odds ratio of having delayed wound healing after last tooth extraction according to steroid use was 4.60 (95% CI, 1.14–18.46) (Table 3). Moreover, the odds ratios for having delayed wound healing after last tooth extraction according to self-reported mild-moderate and severe kyphosis were 4.98 (95% CI, 1.86–13.38) and 2.30 (95% CI, 0.52–10.22), respectively (p for trend = 0.005).

## Discussion

To our knowledge, this is the first study demonstrating the association between experience of delayed wound healing after tooth extraction and self-reported kyphosis. Patients with self-reported mild-to-moderate kyphosis had an approximately 5-fold higher risk of delayed wound healing after last tooth extraction compared with those with self-reported no kyphosis. Our results revealed the possibility that vertebral fracture associated with OP might cause an increased risk of delayed wound healing after tooth extraction. Moreover, as a previous study defined delayed wound healing after tooth extraction as ONJ^2^, OP itself might lead to a risk of ONJ.

Japanese dentists generally worry about performing minor oral surgeries such as tooth extraction, which is considered a potential risk factor for ONJ^15^, because they believe that ONJ is a severe adverse event that cannot be treated. Moreover, Japanese dentists have recently tended to avoid tooth extraction for OP patients who have used BPs and/or other OP agents, such as vitamin D^16^. It has also been reported that delay of tooth extraction due to BP drug holiday increases the occurrence of ONJ^16^. These facts may contribute to the recent rapid increase of ONJ cases in Japan (unpublished data). However, a study demonstrated that >90% of OP patients treated with oral BPs could be cured, whereas 50% of oncology patients treated with intravenous BPs did not show improvement^17^.

Huang *et al*., using data from the National Health Insurance system of Taiwan, concluded that OP patients had a significantly higher risk of ONJ than healthy persons (adjusted hazard ratio 2.05; 95% CI, 1.58–2.65)^12^. Moreover, dental history, such as tooth extraction, may play an important role in the incidence of ONJ, while BPs have a synergistic effect. Conversely, Tennis *et al*. reported that in their sex- and age-matched OP cohort (n = 31,244), the ONJ incidence was 0.26 per 1,000 person-years (95% CI, 0.06–0.47) for patients not exposed to BPs, which decreased to 0.15 (95% CI, 0.00–0.36) after oral BP use^18^. Additionally, Cartsos *et al*. reported that the OR was 0.65 (96% CI, 0.54–0.79) in oral BP-treated OP patients with ONJ in their cohort of 714,217 patients^19^. These results showed that oral BP use may actually decrease the incidence of ONJ. However, the predominant concern in these previous studies is the lack of a strict control group with similar OP characteristics as the patient group. Therefore, studies that categorize patients using BMD values and/or fragility fractures, such as this study, are required.

In this study, patients with self-reported mild-to-moderate kyphosis had an approximately 5-fold higher risk of delayed wound healing after last tooth extraction (p = 0.001). In a previous study, patients who self-reported mild-to-moderate and severe kyphosis had a higher risk of vertebral fractures (OR 2.1; 95% CI, 1.4–3.3 and OR 4.2; 95% CI, 1.8–9.5, respectively)^14^. These findings suggest that subjects with vertebral fractures might have an increased risk of delayed wound healing. However, the lack of a significant association between self-reported severe kyphosis and experience of delayed wound healing may contradict this possibility. The small number of patients with self-reported severe kyphosis may have influenced the results. Additionally, in the crude data (see Table 2), there was no significant difference in age and number of teeth lost between subjects with self-reported no and mild-to-moderate kyphosis, whereas a significant difference was observed in the number of teeth lost between subjects with self-reported no and severe kyphosis. This result was consistent with a recent study^20^. The small number of remaining teeth may result in a decreased risk of infection. It is therefore possible that subjects with self-reported severe kyphosis may have a lower risk of oral cavity infection.

Various risk factors, such as glucocorticoid use, have been reported to affect healing failure after tooth extraction^21^,^22^. In this study, steroid use was significantly associated with an increased risk of delayed wound healing. Almazrooa and Woo described the causes of ONJ as follows: systemic steroids; radiation; bacterial, viral and deep fungal infections; direct chemical toxicity; trauma; avascular necrosis for temporomandibular joint; neuralgia-inducing cavitational osteonecrosis; osteomyelitis associated with sclerotic osseous diseases; and malignancy^21^. Powel *et al*. described that most osteonecrosis cases are secondary to systemically administered corticosteroids and/or high-dose therapy, particularly in patients with underlying comorbidities including connective tissue diseases, hyperlipidemia, or previous trauma^22^. Our finding regarding steroid use is consistent with previous reports. However, a history of diabetes mellitus was not associated with delayed wound healing. Additionally, Huang *et al*. described that the traditional view that people with diabetes have increased risk of delayed healing was not supported in their study^23^. Fernandes *et al*. also reported that although patients with type 2 diabetes mellitus may have impaired neutrophil function, this condition was not associated with an increased risk of experiencing postoperative complications^24^.

It remains unclear whether OP is associated with delayed wound healing after tooth extraction, although OP is associated with the progression of periodontal disease and subsequent tooth loss, especially in postmenopausal women^25^. Using an ovariectomy (OVX) rat model, Zecchin *et al*. concluded that the absence of estrogen may contribute to delayed alveolar wound healing after tooth extraction by interfering with extracellular matrix turnover^26^. Pereira *et al*. also reported that initial molecular changes observed in the absence of estrogen led to delayed alveolar wound healing in OVX rats^27^. Shum *et al*. demonstrated that OP was associated with severe periodontal disease, representing severe clinical attachment loss and interproximal gingival recession, in older Chinese men^28^. Famili *et al*. showed that men with prostate cancer undergoing androgen deprivation therapy were more likely to have periodontal disease than men not such therapy^29^. The etiologies of OP and decreased estrogen and/or androgen concentration might contribute to an increased risk of oral cavity infection.

There are some limitations associated with this study. The primary limitation is that patient responses concerning kyphosis were not obtained at the time of last tooth extraction. Although we selected last tooth extraction to reduce recall bias as much as possible, 347 (67.3%) of all respondents underwent last tooth extraction ≥3 years before the questionnaire survey. Of the 23 subjects who reported mild-to-moderate kyphosis and had experienced delayed wound healing after last tooth extraction, 17 (74%) had undergone tooth extraction more than 3 years previously. Some of these 23 subjects might have had no self-reported kyphosis when their teeth were extracted. Of the five patients who self-reported no kyphosis and had delayed wound healing, three patients underwent extraction more than 3 years previously.

The second limitation is the validity of self-reported delayed wound healing after tooth extraction. It is possible that patients might not associated wound healing failure with dental treatment received. Rather, patients may compare the wound healing status after the last tooth extraction with previous extractions. Additionally, the feeling of problematic delayed wound healing is subjective. However, the lack of significant differences in age, number of teeth lost, and daily number of tooth brushing between subjects with self-reported no and mild-to-moderate kyphosis may suggest little difference in the feeling of problematic delayed wound healing after last tooth extraction.

Ideally, a standard duration of wound healing after tooth extraction is desirable. However, the duration of wound healing after tooth extraction varies among patients according to their underlying conditions. The duration of primary wound healing is typically considered to be within 1 month. Hasegawa *et al*. found that the duration of primary wound healing after tooth extraction in 378 patients who received oral BP therapy was primarily within 4 weeks (94.4% of patients)^30^. Although problematic delayed wound healing after tooth extraction is clinically important, it is unknown whether a patient’s perception of delayed wound healing is associated with prolonged duration of wound healing.

The third limitation is the validity of self-reported kyphosis in men. The association between self-reported kyphosis and risk of having vertebral fractures in women, but not in men, was recently demonstrated^14^. The prevalence of Japanese OP patients aged ≥40 years was 3.4% of men and 19.2% of women^31^. However, the prevalence of patients with lumbar spondylosis was greater among men than women. Self-reported kyphosis is influenced by lumbar spondylosis and spinal degeneration in men. This may contribute to the lack of a significant association between self-reported severe kyphosis and delayed wound healing, although patients with self-reported severe kyphosis had a higher risk of having problematic delayed wound healing after tooth extraction (OR 2.30; 95% CI, 0.52–10.22).

The strength of this study was the comparison of patients with a strict control group using the self–reported kyphosis questionnaire. In previous studies of ONJ, control groups that evaluated the osteoporotic condition were not included. Huang *et al*. concluded that OP patients had a significantly higher risk of ONJ than healthy persons, and BP therapy had a synergistic effect^12^. Although their study did not evaluate OP, the authors showed the possibility that patients treated with BP might have had severe OP accompanied by fractures. Thus, it remains inconclusive whether BP increases ONJ.

In this study, subjects with self-reported mild-to-moderate kyphosis had a significantly higher risk of problematic delayed wound healing after last tooth extraction compared with those with self-reported no kyphosis. Therefore, patients with vertebral fractures associated with OP may be at greater risk of ONJ after tooth extraction. Further studies including patients with concurrent vertebral fractures and delayed wound healing after tooth extraction are necessary to confirm our findings.

## Methods

### Questionnaire-based survey

All patients aged ≥ 50 years who visited our 8 clinics or hospitals from June to August 2014 were invited to complete a structured questionnaire. Patients who had been treated using OP medications (such as BPs) before last tooth extraction, refused to provide written informed consent, and/or had hip fracture when answering the structured questionnaire were excluded from the study. The status of OP medication use was confirmed in each clinic or hospital. Patients who provided many missing or unclear responses were also excluded from the study. The structured questionnaire aimed at collecting the following information: presence of problematic delayed wound healing after last tooth extraction, self-reported kyphosis, number of teeth lost throughout the patient’s lifetime, self-reported periodontal condition, daily number of tooth brushing, history of smoking, history of diabetes mellitus, steroid use, rheumatoid arthritis, and time of last tooth extraction (<1 year, 1–3 years, >3 years). Self-reported kyphosis was defined using three categories as previously described^14^: none, mild-moderate, and severe. Self-reported periodontal condition was divided into two categories: no periodontal symptom and some symptoms like bleeding (with and without brushing), swelling and/or tooth mobility according to our previous study^32^. To reduce recall bias, the presence of delayed wound healing was determined for last tooth extraction.

The Ethics Committee of Matsumoto Dental University reviewed and approved the study protocol. The methods were carried out in accordance with the relevant guidelines. Written informed consent was obtained from all participants.

### Statistical analysis

Continuous variables are expressed as means ± standard deviation (SD). The chi-squared test or one-way analysis of variance (ANOVA) was used to investigate differences in age, gender (women), height (cm), weight (kg), number of teeth lost throughout the patient’s lifetime, self-reported periodontal condition (no or some symptoms), daily frequency of tooth brushing, history of smoking (yes or no), diabetes mellitus (yes or no), steroid use (yes or no), rheumatoid arthritis (yes or no), delayed wound healing after last tooth extraction (yes or no), and time of last tooth extraction (<1 year, 1–3 years, >3 years) among the three self-reported kyphosis categories.

Stepwise logistic regression analysis with forward selection, adjusted for age, gender (women), height (cm), weight (kg), number of teeth lost throughout the patient’s lifetime, self-reported periodontal condition (binary), daily number of tooth brushing, history of smoking (binary), diabetes mellitus (binary), steroid use (binary), and rheumatoid arthritis (binary), was used to calculate odds ratios (OR) and 95% confidence intervals (CI) of delayed wound healing after last tooth extraction according to the self-reported kyphosis category. Data were analyzed using the Statistical Package for the Social Sciences (SPSS, version 22.0; IBM Inc., Chicago, IL, USA). P-values < 0.05 were considered statistically significant.

## Additional Information

**How to cite this article**: Taguchi, A. *et al*. Delayed wound healing after tooth extraction and self-reported kyphosis in Japanese men and women. *Sci. Rep.* **6**, 36309; doi: 10.1038/srep36309 (2016).

**Publisher’s note:** Springer Nature remains neutral with regard to jurisdictional claims in published maps and institutional affiliations.

## Figures and Tables

**Table 1:** Characteristics of 518 study subjects.

	Results
Gender (women)	384 (74.1)
Age (years)	72.6 ± 7.6
Height (cm)	156.2 ± 8.4
Weight (kg)	56.9 ± 9.6
Number of teeth lost	8.4 ± 7.9
Self-reported periodontal condition (some symptoms)	165 (31.9)
Smoking (yes)	167 (32.2)
Daily number of tooth brushing	2.4 ± 1.2
Diabetes mellitus (yes)	54 (10.4)
Steroid use (yes)	14 (2.7)
Rheumatoid arthritis (yes)	27 (5.2)
Problematic wound healing after last tooth extraction	31 (6.0)

Results are given as the mean ± SD or number of subjects (%).

**Table 2:** Characteristics of study subjects according to self-reported kyphosis category.

Self-reported kyphosis category	No	Mild-to-moderate	Severe	p-value
Number of subjects	233	231	54	
Gender (women)	163 (69.5)	182 (78.3)	40 (74.1)	0.075
Age (years)	71.9 ± 7.4^a^	72.6 ± 7.7^b^	75.4 ± 7.8^a,b^	0.011
Height (cm)	157.2 ± 8.7^a^	155.7 ± 7.8	153.8 ± 8.8^a^	0.012
Weight (kg)	58.5 ± 10.5^a,b^	55.9 ± 8.4^a^	54.3 ± 8.9^b^	0.001
Number of teeth lost	7.7 ± 7.7^a^	8.6 ± 7.6	10.7 ± 9.5^a^	0.028
Self-reported periodontal condition (some symptoms)	61 (26.2)	83 (35.9)	21 (38.9)	0.040
Smoking (yes)	91 (39.1)	57 (24.7)	19 (35.2)	0.004
Daily number of tooth brushing	2.4 ± 1.5	2.3 ± 0.8	2.4 ± 1.3	0.692
Diabetes mellitus (yes)	19 (8.2)	25 (10.8)	10 (18.5)	0.078
Steroid use (yes)	4 (1.7)	6 (2.6)	4 (7.4)	0.067
Rheumatoid arthritis (yes)	10 (4.3)	11 (4.8)	6 (11.1)	0.117
Problematic wound healing after last tooth extraction	5 (2.1)	23 (10.0)	3 (5.6)	0.002

Results are given as the mean ± SD or number of subjects (%). The chi-squared test or one-way analysis of variance with Bonferroni correction for multiple comparisons was performed. ^a^p < 0.05; ^b^p < 0.01.

**Table 3:** Odds ratios for having problematic delayed wound healing after last tooth extraction by stepwise logistic regression analysis with forward selection.

		OR	95% CI	p-value
Steroid use (yes)		4.60	1.14–18.46	0.032
Self-reported kyphosis	No	Reference		
	Mild-moderate	4.98	1.86–13.38	0.001
	Severe	2.30	0.52–10.22	0.272

OR: odds ratio, CI: confidence interval.
